# On the Shoulders of Giants: Benefits of Participating in a Dialogic Professional Development Program for In-Service Teachers

**DOI:** 10.3389/fpsyg.2020.00005

**Published:** 2020-02-06

**Authors:** Jose A. Rodriguez, Jose Luis Condom-Bosch, Laura Ruiz, Esther Oliver

**Affiliations:** ^1^Department of Sociology, University of Barcelona, Barcelona, Spain; ^2^Department of Theory and History of Education, University of Barcelona, Barcelona, Spain

**Keywords:** professional development, in-service training, self-efficacy, argumentative skills, dialogic-based training

## Abstract

This study explores the impact of a seminar on self-efficacy and argumentative skills on teachers’ professional development. In this seminar, called “On the Shoulders of Giants,” a group of teachers meet once a month. They debate scientific readings to critically discuss educational theory, which transforms their everyday practices in the school. A survey using a questionnaire was conducted to collect the data. The results show that teachers’ involvement in dialogic-based training positively impacts their ability to address current school problems and that the teachers transfer their new knowledge to their work. The effectiveness of the teachers’ practices increases and, consequently, their students’ learning also improves.

## Introduction

Over the last two decades, international research on professional development has led to an understanding of the factors that contribute to high-quality and effective teacher professional development, which improves teaching and student achievement (Penuel et al., 2007; Pianta et al., 2008; Desimone, 2009; Downer et al., 2009; Higgins and Parsons, 2009; Hill et al., 2013; Knight et al., 2014, 2015; Cordingley et al., 2015; Brown and Weber, 2016; De Naeghel et al., 2016; Jukes et al., 2017; Kutaka et al., 2017). This previous research has identified the positive impact that teacher training programs have on in-service teachers’ activities. According to Desimone (2009), teacher-training programs increase teacher knowledge and skills. Teachers participating in these programs change their attitudes and beliefs and have a great impact on instruction, which increases students’ performance as the main result (Desimone, 2009, p. 185). Highly efficacious teachers tend to be more organized. They try to identify better ways of teaching, drawing on the use of innovative methods that have been supported by scientific research. Borko (2004) also points out the importance of the context for teacher learning. Thus, the educational action that demonstrably produces better results in several different contexts is the so-called dialogic teacher training (Roca et al., 2015).

This article explores a specific bottom-up teachers’ movement, based on recovering the meaning of teacher training through dialogic pedagogical gatherings (seminars) (Roca et al., 2015). These seminars are based on reading and discussing scientific research on educational approaches that successfully improve student outcomes. These seminars have seen exponential growth in attendance in recent years, leading to unprecedented in-service training experiences.

In this article, we present a study of one of these seminars, called “On the Shoulders of Giants”, which adopted the aphorism popularized by Robert Merton to explain scientific progress (Merton, 1965). This study was supported by the European Union’s Seventh Framework Programme for a research project called “IMPACT-EV. Evaluating the impact and outcomes of EU Social Science and Humanities research.”

### Professional Development Research: Impact on Teachers’ Self-Efficacy and Effective Teaching

Over the last two decades, one of the topics in educational psychology research has focused on responding to the need for more empirically validated studies to assess how effectively teachers’ professional development (TPD) improves their self-efficacy, knowledge, skills, and teaching practice as well as how TPD contributes to their personal, social, and emotional growth as teachers. This literature review includes some of these contributions and others from other disciplines that have also provided knowledge about the impact that TPD has on teachers’ and students’ learning (Penuel et al., 2007; Pianta et al., 2008; Desimone, 2009; Hill et al., 2013; Desimone and Garet, 2015; Gutierrez-Cobo et al., 2019; Kurtovic et al., 2019).

Desimone and Garet (2015) suggest five key features on which there is consensus that they make TPD effective: (a) content focus: activities that are focused on subject matter content and how students learn that content; (b) active learning: opportunities for teachers to observe, receive feedback, analyze student work, or make presentations, as opposed to passively listening to lectures; (c) coherence: content, goals, and activities that are consistent with the school curriculum and goals, teacher knowledge and beliefs, the needs of students, and school, district, and state reforms and policies; (d) sustained duration: TPD activities that are ongoing throughout the school year and include 20 h or more of contact time; and (e) collective participation: groups of teachers from the same grade, subject, or school participate in TPD activities together to build an interactive learning community (Desimone and Garet, 2015, p. 253).

In recent years, TPD research has focused on creating knowledge that contributes to teacher quality and effective teaching in a context in which students are increasingly diverse, guaranteeing both equitable learning opportunities and outcomes (Knight et al., 2015; Impedovo, 2016; Chatelier and Rudolph, 2018). The latest contributions are directed toward reconceptualizing high-quality teacher professional development, starting from a socially inclusive pedagogic work based on what is already known about the new conceptions of teaching and learning that are contributing to not leaving any students behind (Knight et al., 2015; Gale et al., 2017; Lampert et al., 2019).

In this context, TPD research has increased considerably and has focused on evaluating the impact that in-service TPD programs have on both teacher learning and student achievement in mathematics, language, literacy, reading, science, and arts from early childhood education onward (Downer et al., 2009; Higgins and Parsons, 2009; Brown and Weber, 2016; Caddle et al., 2016; De Naeghel et al., 2016; Jukes et al., 2017; Kutaka et al., 2017; Shernoff et al., 2017). Some of this research has further focused on the learning experiences that promote teachers with professional development programs that are based not only on best practices but also on practices that reflect their students’ sociocultural context (Brown and Weber, 2016). These practices also start from solidarity with families and local communities to guarantee professionally developed teachers who respond to their students’ needs (Zeichner et al., 2016; Lampert et al., 2019).

There is also a significant amount of literature on TPD regarding evidence-based approaches that promote interaction, dialogue, and reflection in the classroom, that achieve positive results regarding children’s literacy (Pianta and Hamre, 2009), and that create productive learning environments in classrooms (Hagelskamp et al., 2013). Within the framework of this research, some analyses pay attention to the need for TPD to be aligned with research on effective teacher professional development so that TPD has an impact on the self-efficacy of teachers and the improvement of student outcomes (McElearney et al., 2018).

Nevertheless, some contributions have provided relevant insight into this field from different perspectives and disciplines. Some of these contributions have been realized in qualitative case studies on the effectiveness of TPD in integrating interdisciplinary civic education into high school humanities courses (Barr et al., 2015).

Specifically, the TPD assessed by Barr and colleagues was focused on the development of workshops, in which teachers are engaged in reflective discussions on evidence-based approaches to teaching interdisciplinary historical case studies. This program’s assessment produced important evidence regarding the progress of teacher self-efficacy. This assessment also revealed important steps regarding student learning achievement, such as the building of historical thinking and civic dispositions, as well as the capacity to engage in relevant ethical reflections (Barr et al., 2015). Five elements of success were identified that could be integrated into other professional development programs: (1) active learning as an opportunity to be involved instead of passive learning as a mere attendee, (2) coherence between theory and practice, (3) connection to the demands placed on teachers, (4) ability to apply the knowledge gained in schools in which the teachers are involved, and (5) collective participation based on teachers’ involvement in reflective practices, in which evidence-based approaches to TPD and teaching are discussed (Barr et al., 2015). The last dimension has been widely explored as a successful component of TPD (Marques et al., 2016; Paula, 2018).

There is strong evidence on how the dialogue and joint reflection of teachers on pedagogical content improves their self-efficacy. Kiemer et al. (2018) analyzed the impact of a TPD program on productive classroom discourse and communication strategies on teacher practice. Ten teachers from science and mathematics classes from middle and high schools participated for a whole year in various theoretical seminars on productive classroom discourse and the scaffolding of student ideas. After the theoretical inputs, the teachers reflected together on how knowledge about high-quality discourse and active learning methods could enrich their classes. Approximately 2 weeks later, after the theoretical workshops, the teachers were recorded in their lessons in what has been called “dialogic videos.” Afterward, a selection of these videos was viewed by the teachers. Again, the teachers discussed these together, but this time, they placed their professional development in a realistic context. After participation in this TPD program, all teachers reported that they had changed their teaching practice. They began to use a discourse that motivated students to learn and participate in the classrooms. In turn, the students perceived their teachers as more competent in fostering a supportive context for students and in promoting a climate of motivation for learning.

Along these lines, other studies have explored the importance of including the active agency of teachers in their professional development as a mechanism to improve their self-efficacy (Pyhältö et al., 2015; Tam, 2015; Wanless et al., 2015; Ciampa and Gallagher, 2016; Liu et al., 2016). For instance, Liu et al. (2016) analyzed the impact of 6 years of the TPD program on building a self-sustainable and democratic community of practice. On several Saturdays during these school years, workshops were held that combined lectures, readings, and discussions on East Asian topics according to the Wisconsin K-12 Social Studies Content Standards. This study identified that ongoing teachers’ dialogue and reflection on the application of content in their classes not only improved their knowledge and skills to develop new ways of internationalizing their curricula and schools but also improved their ability to research, organize, and lead initiatives in their schools and communities.

Finland is one of the countries promoting TPD based on scientific evidence that has been shown to improve the self-efficacy of teachers and student learning. One of the TPD studies developed in Finland was carried out with a survey of all comprehensive schoolteachers. This study identified that one of the keys to an effective TPD was the promotion of dynamics that would facilitate the active participation of teachers in their professional development. The results indicate that effective TPD consists of several elements, including skills, efficacy beliefs, and motivational factors, which entail transforming one’s teaching practices, experiencing collective efficacy, constructing positive interdependency, appreciating mutual agreements, and using active strategies for help-seeking (Pyhältö et al., 2015).

Teachers’ professional development that is based on a collaborative inquiry is another approach that leads to effectiveness and that has an impact on teachers’ self-efficacy (Ciampa and Gallagher, 2016). This approach creates opportunities for teachers to become involved in building pedagogical knowledge together by means of a permanent dialogue through the following four stages: (1) identifying the problem, (2) collecting evidence, (3) analyzing evidence, (4) and reflecting, sharing, and celebrating the results. Ciampa and Gallagher (2016), in their qualitative case study with elementary (grade 8) and secondary (grade 9) literacy teachers who participated in a TPD program on the collaborative inquiry approach, identified that teachers improved their skills in understanding common student literacy learning needs and effective literacy instruction and assessment practices.

Furthermore, there is also evidence that in TPD programs that promote effective and active teachers’ involvement teachers not only improve their learning but also assume a responsibility for the professional growth of their colleagues, thus transforming their communities of practice into a workforce of teachers who are committed to creating successful schools (Tam, 2015).

Another effective TPD approach identified is the “responsive classroom approach” (Wanless et al., 2015). Attention is paid to the implementation of evidence-based interventions that are aimed at achieving safe, challenging, and joyful classrooms and schools by combining social and academic learning for all children. This approach stresses the importance of developing teachers’ emotional skills to manage classroom social relationships and encourage a proper climate in parallel with students’ academic achievement (Wanless et al., 2015).

However, evidence also exists that professional communities become spaces where productive learning to reach the aforementioned objectives is not commonly undertaken (Opfer and Pedder, 2011; Popp and Goldman, 2016). For this reason, the impact of professional development programs must be assessed according to teachers’ self-efficacy and their impact on improving student performance, and research must be conducted on the features of effective and ongoing professional development that promote a disposition in teachers toward a lifelong evidence-based learning (Knight et al., 2015).

## How the Seminar “On the Shoulders of Giants” Works

The seminar “On the Shoulders of Giants” started in 2012 through the initiative of 20 primary and secondary school teachers in the city of Valencia, Spain. The teachers started gathering relevant research contributions that provided relevant knowledge on which educational actions improve children’s learning. This seminar is not compulsory; teachers participate voluntarily in the training, which is self-organized on Saturdays, once a month, outside of regular working hours. Nonetheless, after 6 years, this seminar is established and sustainable, as more than 100 teachers are currently registered. Furthermore, an average of 80 teachers regularly attend the seminar every month.

Participants are preprimary, primary, and secondary school teachers, including principals and educational advisors from different cities and towns in the Autonomous Community of Valencia, Spain. At the beginning of the academic year, books and scientific articles are selected for discussion in each session. Some authors that these teachers have read and discussed include Lev Vygotsky, Jerome Bruner, and Paulo Freire, among others. Additionally, the latest contributions to learning and teaching published in international peer-review journals with high scientific impact factor are also discussed.

This new movement has been defined as dialogic teacher education (Roca et al., 2015), in which teachers are exposed to scientific reasoning regarding teaching, learning, and educational actions that have previously been implemented and that have achieved relevant learning outcomes. In dialogic teacher education, changes arise when teachers’ professional development is based on scientific evidence and there is an acknowledgment that teachers and students’ families have the right to obtain the best and latest international knowledge about learning and educational research (Garcia-Carrion et al., 2017). The seminar “On the Shoulders of Giants” is an example of such an approach.

Participants read the literature before each session and select at least one paragraph of interest to share with the group. Each Saturday, the seminar proceeds as follows: between 9:30 and 11 am, all participants discuss the selected reading according to the principles of an egalitarian dialogue with respect to different opinions. Based on these criteria, anyone who wants to speak raises his/her hand and develops an argument based on a passage from the selected article or book, which should be referenced by page and paragraph. Thus, other participants can add comments regarding the same passage to collectively provide a deeper reflection on their teaching practice. At 11:30 am, after a coffee break, participants split into several thematic working groups in which desired goals for the year are shared and agreed upon. These groups mainly discuss and share the impact of evidence-based actions that participants are implementing in their classrooms and schools. These teachers achieve the double task of finding and reviewing the scientific literature (to expand their knowledge and propose readings to the entire group) and sharing experiences regarding the implementation of the evidence-based actions discussed. At the end of the academic year, a closing session is held, consisting of a 1-day session in which the participants are invited to evaluate the seminar and draw conclusions for the upcoming year. Participants in this seminar use Telegram, Google Drive, Facebook, and Twitter to organize, coordinate, and share documents and materials. Using these tools, the participants produce leaflets, notes, and papers that are made available to all members. Most participants also use social networks to spread information and receive updates, thereby creating a network of professionals who not only share a monthly space but also become a reference group for the teaching profession.

## Research Method

### Research Question and Survey Instrument

The research question addressed in this study is as follows: Which are the impacts or benefits that teachers report as a consequence of their participation in the seminar “On the Shoulders of Giants”?

The data used in this research were derived from a questionnaire (see Supplementary Material) aiming at analyzing the aspects that motivate the participating teachers in the seminar “On the Shoulders of Giants” held in Valencia, as well as at analyzing the benefits of this participation. The questionnaire was distributed among preprimary, primary, and secondary teachers both at the end of the journey and through a digital platform. A total of 40 items were included in the questionnaire, organized into seven sections: context, the functioning of the seminar, satisfaction with the seminar, the usefulness of the seminar and empowerment, intention to continue attending the seminar, relationships, and sociodemographic information. The results presented in this article are related to the specific questions that focused on the teachers’ perceptions of how the readings and discussions in the seminars are useful for improving teachers’ self-efficacy and argumentation skills in their professional practice and how they are useful for improving students’ achievement. The questionnaire was designed *ad hoc* for implementation at a particular seminar session, in which it underwent pilot testing with a small number of participants to correct potential bias items. The questionnaire included multiple-choice items as well as items based on the use of a Likert scale ranging either from 1 (“totally disagree”) to 10 (“totally agree”) or from 1 (“minimum”) to 10 (“maximum”).

### Sample

The seminar “On the Shoulders of Giants” was defined as a case study. Purposive sampling was used, asking teachers who attended the seminar “On the Shoulders of Giants” to participate voluntarily in the survey. A total of 69 preprimary, primary, and secondary teachers decided to participate. A total of 69.6% of the participants in the survey were women, whereas 30.4% were men (which is in line with the regular gender distribution of teachers in Spain). Half of the teachers were between 30 and 40 years old (51%), while 30.3% were older than 40 years old (between 41 and 50), and almost two teachers over 10 were “young” teachers with “limited” teaching experience (18.7%). The quantitative case study, therefore, aims to evaluate the distribution of the variables and the relationships between them. The study cannot be extrapolated to other cases because it is not a comparative study, but this fact does not limit or invalidate its results (Yin, 2018).

### Procedure

The questionnaire was distributed in paper format to all seminar attendees at the end of a Saturday meeting, as well as in digital format through a digital platform. Ethical procedures were presented to the participants. Researchers explained the objectives of the study, noting the potential risks of participating in the survey, the contact information for further questions about the project, and the explicit condition that participation is voluntary. An open space was set up for the participants to ask questions and resolve their doubts regarding the survey. Participants were also informed both during the seminar and on the digital platform that any data collected would be kept anonymous and confidential. Participants provided either oral or digital consent (agreeing to answer the questionnaire) to participate in the survey. All participants were informed about their right to leave the survey at any time and remove their data from the database. The study was approved by the Ethics Committee of the Community of Researchers on Excellence for All (CREA).

### Data Analysis

A descriptive statistical analysis was conducted on the data collected from the survey to answer the research question. Additionally, multivariate analysis was conducted to explore potential associations among variables, drawing on the use of the correlation coefficient in the bilateral analysis. Several categorical analyses were established about teacher participation in the seminar and the learning and self-efficacy improvements examined. Drawing on the sets of questions comprising this questionnaire (contextualization, dynamics of the seminar, satisfaction with the seminar, seminar utility, and empowerment), the following three main statements framed the data analysis: (1) scientific knowledge leading to an educational improvement, (2) strengthening debate skills with colleagues, and (3) improving teaching practice and student learning. In terms of the validity of the content, a confirmatory analysis with experts was conducted. Regarding the validity and reliability of the questionnaire, a factorial analysis was conducted.

## Results

The data collected suggest that participating in the seminar “On the Shoulders of Giants” not only impacts teachers’ practices and attitudes toward in-service teacher training but also encourages “networking” among teachers, who are more willing to create or participate in collaborative discussions, drawing on the principles of dialogic learning (Flecha, 2000) and defining what we call “dialogic teacher education”. Therefore, according to the respondents of the survey, the benefits of participating in the seminar may include four different types:

• Transforming the individual teachers’ practices.
• Increasing the use of scientific evidence to inform teachers’ practices.
• Engaging in and creating networks of teachers for discussing lesson plans and practices with colleagues, drawing on scientific evidence.
• Noticing students’ improvement in terms of learning.

### Transforming the Teachers’ Practices

“On the Shoulders of Giants” is distinctive because it is based on reading and discussing scientific literature on evidence-based approaches to teaching and learning. We found a significant correlation between involvement in the seminar and the use of the scientific knowledge acquired (see Table 1). Spearman’s bivariate analysis revealed a high correlation between involvement (number of articles/books read) and the use of scientific knowledge.

**TABLE 1:** Spearman’s bivariate analysis of involvement in the seminar and use of scientific literature.

		**Provides key**	**Helps identify**	**Provides access to**	**Provides a network of**
		**information to**	**best solutions**	**other forums (seminars,**	**professionals who I**
		**understand current**	**based on scientific**	**conferences, etc.) that**	**can rely on and collaborate**
		**school problems**	**evidence**	**are very useful for**	**with to improve education**
				**improving my practice**	
Involvement (books read)	Correlation coefficient	0.497*	0.447*	0.582*	0.522*
	Sig. (bilateral)	0.002	0.006	0.000	0.001
	N	36	37	37	37

*Correlation is significant at the 0.01 level (bilateral).

According to the respondents of the survey, participating in the seminar provides them with access to other forums (seminars, conferences, etc.) that might help expose them to data, practices, and other sources of knowledge that they consider “very useful” for improving their practice. Checking other sources of evidence, participating in networks of professionals, and using scientific evidence as the main source of “evidence” appears to transform these teachers’ regular practices. The data suggest that these three variables are somehow associated with the practice of participating in the seminar (Table 1).

### Increasing the Use of Scientific Evidence to Inform Teachers’ Practices

The abovementioned data indicate a clear increase in the use of scientific knowledge that is associated with attending the seminar. Attending the seminar greatly contributes to increasing the number of scientific books and journal articles read by the teachers (see Table 2 for a comparison of before and after the respondents’ participation in the seminar).

**TABLE 2:** Use of scientific literature by teachers.

**Number of scientific**	**Before participation**	**After participation**
**articles/books read**	**in the seminar**	**in the seminar**
None	28.4%	0.0%
Between 1 and 5	44.8%	21.7%
Between 6 and 10	9.0%	29.0%
Between 11 and 20	6.0%	17.4%
More than 20	11.8%	31.9%
Total	100.0% (*n* = 67)	100.0% (*n* = 67)

Furthermore, we found that for teachers involved in this seminar, the improvement in professional practice meant not only methodological improvements but also improvements in the learning outcomes of their students as well as improvements in the school and classroom climate (74.5%) and the effectiveness of the methodologies implemented (72%) (see Table 6).

Nearly 29% of the participants never read a scientific journal article on educational research and learning processes before attending this seminar. After becoming involved in the seminar, that practice was dramatically transformed: no one declared not reading any scientific articles or books. On the contrary, three out of four teachers participating in the survey claimed that they had read six articles/books or more (31.9% of participants said that they had read more than 20 articles or books). This is a significant transformation of teachers’ practices, especially because most of them come from a tradition of not reading scientific literature related to their professional activity. These findings also show how the participation in this professional development activity was a turning point for a large number of participating teachers regarding the use of scientific literature to understand current school problems, identify best solutions, and improve their practice.

### Engaging in and Creating Networks of Teachers to Discuss Scientific Research Evidence to Improve Their Teaching Practices

Most of the teachers agreed that they acquired new scientific knowledge and that they corrected their pre-existing assumptions through the seminar readings (see Table 3). This finding is particularly relevant because unlike other fields of social action (such as health), many education professionals in Spain base their teaching strategies on popular beliefs or assumptions and not on evidence-based practices (Flecha, 2015).

**TABLE 3:** Knowledge acquisition by teachers after participating in the seminar.

**Question**	**% Answers**
Acquired new knowledge about the scientific basis of learning that I did not know about in the past	89.9% (*n* = 62)
Acquired new knowledge about the scientific basis of learning that corrected preexisting assumptions about learning	73.9% (*n* = 62)
Strengthened and extended preexisting knowledge	58.0% (*n* = 62)

By participating in the seminar, many teachers developed the ability to improve their practices using solid information about proven key elements of learning from fields such as psychology, pedagogy, and sociology. Moreover, when the teachers were asked whether discussing these readings during the seminar helps them strengthen their capacity for analysis and debate with other education professionals, 98.6% answered “yes”. This newly developed capacity for argumentation can be explained by the high rate of participants who use scientific knowledge to understand educational problems aimed to find the best solutions to overcome these problems (see Table 4).

**TABLE 4:** Impact of the seminar on the use of scientific knowledge for debating with other professionals.

**Question**	**(%) Scale from 1 to 10**					
	**1 to 4**	**5 to 7**	**8 to 10**	**Total**	**Mean**
It provides me with key information to understand current school problems	1.5%	19.4%	79.1%	100.0% (*n* = 67)	8.493
It helps me identify the best solutions according to scientific evidence	1.5%	29.4%	69.1%	100.0% (*n* = 68)	8.353

The seminar became a space for teachers to network (Table 1) based on a discussion of selected articles and books and to collaborate to improve their practices.

### Improving Teaching Practice and Student Learning

**FIGURE 1:**
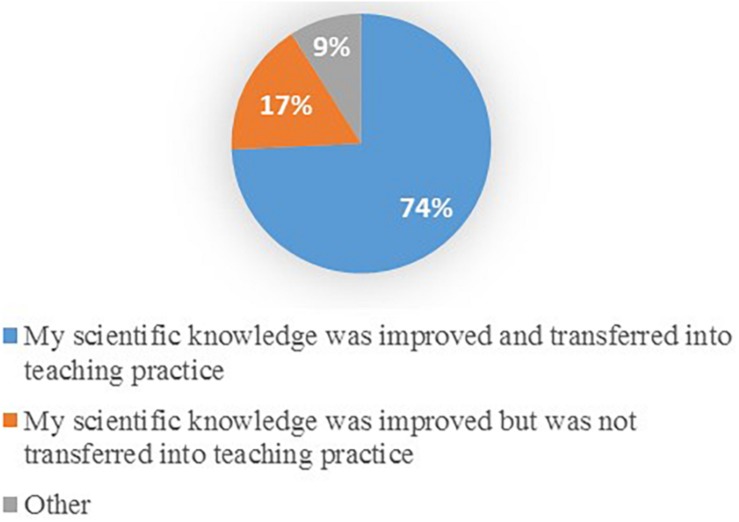
Impact of the seminar on teaching practice.

The impact of the seminar on the improvement in teaching practice and student learning was found to be one of the primary benefits of participation in the seminar. However, this study further shows the real impact on the teachers’ professional practice. Figure 1 and Table 5 show that most of the teachers (74.2%) transferred their learning of evidence-based educational actions to their teaching practice.

**TABLE 5:** Impact of the seminar on improvement in teaching practice.

**Question**	**% Answers**
Both my scientific knowledge and my teaching practice improved because I transfer the knowledge acquired to my work	74.2%
While my scientific knowledge is improving, I still find it difficult to translate this knowledge into my teaching practice	16.7%
It did not improve my knowledge or my teaching	0%
I do not identify with any of these statements	1.5%
Other	7.5%
Total:	100% (*n* = 68)

In the questionnaire, teachers evaluated the impact of their training on student performance using a score between 1 and 10, where 1 represented the minimum and 10 represented the maximum. Most of the teachers responded with scores between 8 and 10, especially regarding learning (64.7%), attention (65.3%), and the capacity for reflection and debate (83.7%). Evidence of improving students’ achievement after implementing evidence-based educational actions was very relevant for teachers who participated in this study and played a key role in their continued interest in attending the seminar.

## Discussion

Over the last decade, the research has claimed that TPD has a great impact on improving in-service and preservice teachers’ practices in the classroom. International research on teacher professional development has already shown that teachers attend professional development courses to improve their self-efficacy in their professional practice (Gorozidis and Papaioannou, 2014, 2011). This is also the case with this seminar. Evidence suggests that teachers with a solid academic background are more able to adjust their teaching based on informed and systematic reflection, which has a great impact on students’ performance. Thompson (2012) compared the achievement of students who had teachers with an extensive academic background and found that teachers with professional training correlate positively with students’ performance. Shulman (1986) coined the term pedagogical content knowledge (PCK) to refer to this type of a background, adding the pedagogical component to “classic” content knowledge, which has been at the center of the TPD programs. An extensive body of research has provided evidence of TPD impact on teachers’ practice (Pianta and Hamre, 2009; Barr et al., 2015; Liu et al., 2016; Matic, 2019). The results reported by the participants in this study are consistent with this previous body of research. The teachers participating in the seminar “On the Shoulders of Giants” claim that attending the seminar provides them with solid training to address “current school problems” effectively, helping them identify the best solutions to solve these problems. The five features identified by Desimone and Garet (2015) that make TPD effective are also present in the teachers’ discourse in the survey discussed here. The teachers highlight aspects such as content (drawing on their readings), active learning, and collective participation, which is consistent with Desimone and Garet’s (2015) findings. TPD provides in-depth training for in-service teachers, who become more able to “identify the best solutions based on scientific evidence” (Table 1).

As in these previous studies, the survey conducted in this study confirms that TPD contributes to improving teachers’ practices. However, unlike these previous studies, the case of the seminar “On the Shoulders of Giants” suggests that TPD might be based on “scientific evidence”, that is, the use of evidence that has been confirmed by the scientific community (i.e., contributions of authors such as Vygotsky, Bruner, Freire, Flecha, etc.). Barr et al. (2015) point out that “workshops with teachers” contribute to engaging the teachers in a reflective discussion, whereby the teachers develop the agency to make critical decisions in everyday school-learning situations. Similarly, the teachers participating in the seminar “On the Shoulders of Giants” claim that discussing current problems in light of others’ contributions (connecting practice with theory) (a) provides key information for understanding current school problems and helps them find the best solutions (drawing on research-evidence from previous cases). Teachers increase their knowledge of these “scientific bases” by reading classic books (in education, the psychology of education, and other related fields). The data show how the teachers’ participation in the seminar greatly increased the number of scientific articles and books read by the teachers who participated in the survey (Table 2). This practice not only (a) transformed the teachers’ practices (as teachers) in terms of in-service TPD activities but also (b) became the basis for their actions in the school.

The dialogic teacher training illustrated by the seminar “On the Shoulders of Giants” is consistent with the findings of Barr et al. (2015): “the ability to apply the knowledge gained in schools” with the added component of “dialogic,” as at type of reflective practice based on the use of dialogic speech (Garcia-Carrion and Diez-Palomar, 2015) to discuss the chosen readings. Drawing on dialogic speech, participants in the seminar can understand the contributions of the previous research in teaching and related learning fields, providing them with key information to understand current school problems (Table 2). Expanding teachers’ backgrounds through their participation in the seminar also explains the transformation of their practices, because a high number of the teachers tends to transfer the new knowledge acquired into their work (Table 5).

The data collected are also consistent with Barr et al. (2015), who found that “collective participation in reflective practices” is one of the main ways for teachers to improve the effectiveness of their practice. There are different approaches based on this finding that have already been validated by research, such as lesson study (Murata, 2011; Fernandez and Yoshida, 2012) and the use of didactical artifacts such as suitability criteria (Vanegas et al., 2014). Teachers participating in the seminar “On the Shoulders of Giants” also develop a sense of networking, sharing a dialogic reading that opens space for a multiplicity of entry points into the readings and that results in a deeper understanding (which is also consistent with relevant findings from research in psychology, which has found that cognition is socially constructed). Table 6 reports the extensive impact (in terms of learning, attention, interest, reflection, etc.) that participation in the seminar has on the teachers.

**TABLE 6:** Impact of the seminar on student improvement.

	**(%) scale from 1 to 10**						
	**% answers**	**1 to 4**	**5 to 7**	**8 to 10**	**Total**	**Mean**
Improved learning	73.9%	0.0%	35.3%	64.7%	100.0%	7.941
Improved attention and interest	75.4%	0.0%	34.7%	65.3%	100.0%	8.096
Improved reflection and debate	71.0%	0.0%	16.3%	83.7%	100.0%	8.673
Improved effectiveness of the methodologies implemented	72.5%	0.0%	28.0%	72.0%	100.0%	8.300
Improved classroom climate	73.9%	0.0%	25.5%	74.5%	100.0%	8.196
Improved school climate	68.1%	6.4%	34.1%	59.5%	100.0%	7.617

## Conclusion

This study contributes to the field of teacher professional development by providing new insights into the role of teacher training programs in in-service teachers’ practices. As discussed above, scientific evidence-based literature has become a strong reference to improve both teachers’ knowledge backgrounds and practices. Drawing on this kind of literature, the teachers participating in this study noticed benefits for their students’ learning. At the same time, the teachers recognized improvements in their own analytical and argumentative skills while interacting with other education professionals who are consistently connected to scientific evidence for student improvement. Therefore, science-based in-service professional development strongly empowers teachers in their professional practice.

Based on this study, teachers attributed the improvement in their professional practice to their participation in the “On the Shoulders of Giants” seminar. Additionally, the teachers related this improvement to improvements in their students’ school performance. All of these motivations are enhanced by the idea of stepping “On the Shoulders of Giants,” referring to the scientific knowledge acquired in this seminar. Participating in this seminar provided teachers with the scientific skills needed to better understand the realities that schools are currently facing. Moreover, the teachers were better prepared to find possible solutions based on scientific evidence.

The seminar “On the Shoulders of Giants” is an example of a dialogic evidence-based in-service professional development space aimed at social impact. Teacher professional development that draws on scientific literature leads to actions that improve school performance and learning outcomes. This study focuses on one teacher professional development initiative that is voluntarily attended after working hours, with no credit awarded for attendance. The main findings highlight the seminar’s social impact and how professional training and practice are linked to acquiring the knowledge and strategies needed to improve student results, thereby transforming students’ future opportunities. It is necessary to point out the limitations of the results. The findings could be further enriched through interviews with teachers to obtain more details not only on the benefits of their participation in the seminar but also on the aspects of the seminar to be improved. Further research on this TPD initiative is necessary for a more in-depth evaluation of its social impact: for instance, by performing qualitative research using in-depth interviews with and narratives of participating teachers, families, and students.

## Data Availability Statement

The datasets generated for this study are available on request to the corresponding author.

## Ethics Statement

The studies involving human participants were reviewed and approved by the Ethics Committee of the Community of Researchers on Excellence for All (CREA), University of Barcelona. The patients/participants provided their written informed consent to participate in this study.

## Author Contributions

JR and JC-B designed the fieldwork and participated in the data analysis and drawing of the conclusions. LR and EO participated in the data analysis and wrote the final version of the manuscript.

## Conflict of Interest

The authors declare that the research was conducted in the absence of any commercial or financial relationships that could be construed as a potential conflict of interest.
