# Supplementary material for: On the Shoulders of Giants: Benefits of Participating in a Dialogic Professional Development Program for In-Service Teachers
Source: Front Psychol. 2020 Feb 6;11:5. doi: 10.3389/fpsyg.2020.00005 (PMC7025525; doi:10.3389/fpsyg.2020.00005)
Supplement: Supplementary file 1 [file Data_Sheet_1.pdf]

Supplementary Material

Appendix: Questionnaire. Teachers Development through Scientific Training

Section 1. Contextualization

1. You found out about the Valencia Seminar through...

Friends who told you about it
Colleagues from your school
Educational forums such as congresses, seminars, conferences, etc. (specify):
Internet (specify):
Other (please specify):

2. How long have you been participating in the Seminar? (Information: the Valencia Seminar started in February 2012)

Since its inception in February 2012
More than 3 years
More than 2 years
More than 1 year
Less than 1 year

3. What were the main reasons that made you decide to participate in the Valencia Seminar? (You can mark several options with an X)

Meet people
Learn more
Improve professional practice
Reengagement with the profession
Other (please specify):

Section 2. Seminar Dynamics

4. On a scale of 1 to 10 (where 1 is the minimum and 10 the maximum), what degree of involvement do you think you have in each of the following activities of the Seminar?

Attendance:
Oral interventions:
Presentation of chapters:
Dissemination of the seminar to other colleagues, friends, acquaintances, etc.:
Logistics:
Accompaniment (e.g., when new people join):
Translation:
Other (specify a value from 1 to 10):

5. On a scale of 1 to 10 (where 1 is the minimum and 10 is the maximum), how much organizational effort is required for you to attend the Valencia Seminar regularly?

Family:
Work:
Personal:
Other (specify a value from 1 to 10):

6. What kind of works do you read and discuss in the Seminar? (You can mark several options with an X)

Any book of interest to the Seminar participants
Books by authors of international scientific relevance
Articles published in scientific journals included in the main international rankings
Other scientific articles
Articles published in nonscientific journals
Press articles
Other (please specify):

7. How many scientific books and/or articles have you read before participating in the Seminar?

None
Between 1 and 5
Between 6 and 10
More than 11
More than 20

8. How many scientific books and/or articles have you read at the Seminar?

None
Between 1 and 5
Between 6 and 10
More than 11
More than 20

Section 3. Satisfaction with the Seminar

9. On a scale of 1 to 10 (where 1 is the minimum and 10 the maximum), what degree of personal and professional satisfaction does attending this Seminar regularly give you?

10. On a scale of 1 to 10 (where 1 is the minimum and 10 is the maximum), to what extent have your expectations in this regard been met?

Section 4: Seminar Utility and *Empowerment*

11. Since participating in the Seminar (you can mark several options with an X),

You have acquired knowledge about the scientific basis of education that you did not know about in the past.
You have deepened and solidified preexisting knowledge on the scientific bases of education.
You have acquired new knowledge about scientific bases of education and you have understood knowledge acquired in the past that you had not interpreted correctly.

12. Which of these statements do you identify with to assess the impact that the Seminar has on the improvement in the education that you provide to your students?

It has improved my training and the content of my teaching because I transfer the knowledge and reflections acquired in the Seminar to my teaching work.
Although my scientific training is improving, I still find it difficult to transfer the content acquired in the Seminar to my teaching in the classroom.
It has not improved my training or the content of my teaching.
I do not identify with any of these statements.
Other (please specify):

If you have indicated **the first of the options,**

12.1. On a scale of 1 to 10 (where 1 is the minimum and 10 is the maximum), assess the impact of your participation in the Seminar on the students in the following fields:

Improved learning:
Improved attention and interest:
Improvement in reflections and debates:
Improvement in the effectiveness of the methodologies used:
Improvement in the coexistence in the classroom:
Improvement in the coexistence outside the classroom:
Other (specify a value from 1 to 10):

13. In discussions with other colleagues (teachers or other education professionals) on issues related to educational practice, do you think that participating in the Seminar has improved your analysis and arguments about the educational reality?

Yes
No
If **yes,**

13.1 On a scale of 1 to 10 (where 1 means "in total disagreement" and 10 means "completely in agreement"), rate each of the following statements on how such an improvement is reflected:

It gives me key information to understand the problems that schools face:
It helps me analyze each concrete situation and identify the best solutions based on scientific evidence:
The debates generated around the scientific works we read are a very important source of information to improve educational actions:
The Seminar gives me access to other educational debate forums (congresses, conferences, seminars, etc.) that are very useful for improving my work:

The Seminar provides a network of teachers and education professionals who I can count on at different times to resolve my doubts and carry out new initiatives for educational improvement:
Other (specify a value from 1 to 10):

14. If you have children, do you think that your participation in the Seminar has or has had an impact on your children's education?

Yes
No

14.1. On a scale of 1 to 10 (where 1 is the minimum and 10 is the maximum), rate the following items:

Improved communication with me:
Improved interest in my work:
Improved interest in their education:
Improvement in their thinking:
Improvement in family cohabitation:
Improved relationships with peers:
Other (specify a value from 1 to 10):

15. Has belonging to/the support of your fellow seminar participants allowed you to do any of the following during the past year? (You can mark several options with an X)

To select a project you found interesting
To make a proposal that you considered appropriate
To support or oppose a proposal submitted by others
To try new ways of doing things
To undertake changes in the institution
Nothing

16. On a scale of 1 to 10 (where 1 means "in total disagreement" and 10 means "completely agree"), rate your participation in the seminar according to the following statements:

My participation in the Seminar has increased my motivation to work in the field of education:
My participation in the Seminar allows me to improve my professional practice:
My participation in the Seminar allows me to be successful in my professional projects:
My participation in the Seminar helps me feel happier and more satisfied both professionally and personally:

Section 5. Intention to Continue

17. Do you plan to continue participating in the seminar?
Yes
No

18. What are currently your main reasons for continuing to participate in the Valencia Seminar? (You can mark several options with an X)

Meet people
Learn more
Improve professional practice
Reengage with the profession
The feeling of community that the Seminar provides
Maintain connections with the group
Other (please specify):

Section 6. Relations

The objective of this part of the questionnaire is to obtain information on the relations among the members of the Seminar and their environment, which allows the construction of organizational maps, the evaluation of the impact of the relations on the success of the project, and the construction of individual and collective strategies to maximize the profitability of the relational activity. This information is intended to broaden the range of resources of the Seminar members to evaluate (and improve, where appropriate) the processes of individual and collective action.
To carry out the project, it is necessary to complete the questionnaire, and personal identification is essential. Given the characteristics of the information provided, the data will be treated anonymously and with the utmost confidentiality. The results will, of course, be completely confidential and the names of the persons will not appear or be identifiable under any circumstances.
Using the attached listings of the Seminar, answer the following questions on relations by indicating the appropriate persons (those with whom the relationship is established or referred to) and by using (whenever possible) the code associated with the person. In the case of persons or institutions that do not appear in the listings, write their names as completely as possible.

PLEASE INDICATE YOUR IDENTIFICATION NUMBER FROM THE ATTACHED LIST:

19. With whom do you speak the most about the Seminar projects?

Members of the Seminar (write the number corresponding to the people to whom you are the most related from the attached list):
People and/or institutions outside the Seminar that you have begun to engage with or attend as a result of your participation (use the following table to indicate the initials, position, and institution of the five people to whom you are related the most):

Name (initials only)
Position
Institution

20. With whom do you collaborate on projects/activities related to the Seminar?
Members of the Seminar:
People and/or institutions outside the Seminar that you have begun to engage with or attend as a result of your participation:
Name (initials only)
Position

Institution

21. Who do you ask or consult about issues related to the Seminar?

Members of the Seminar:
People and/or institutions outside the Seminar that you have begun to engage with or attend as a result of your participation:
Name (initials only)
Position
Institution

22. Who do you turn to for help or ideas and suggestions to solve a problem?

Members of the Seminar:
People and/or institutions outside the Seminar that you have begun to engage with or attend as a result of your participation:
Name (initials only)
Position
Institution

23. Who have you helped with issues related to the Seminar?

Members of the Seminar:
People and/or institutions outside the Seminar that you have begun to engage with or attend as a result of your participation:
Name (initials only)
Position
Institution

24. Who helped you with matters related to the Seminar?

Members of the Seminar:
People and/or institutions outside the Seminar that you have begun to engage with or attend as a result of your participation:
Name (initials only)
Position
Institution

25. With whom do you have a good friendship?

Members of the Seminar:
People and/or institutions outside the Seminar that you have begun to engage with or attend as a result of your participation:
Name (initials only)
Position
Institution

26. Do you know the following people personally or can you access them through an acquaintance, a friend or a family member? *(Mark all possible options with an X)*

	I know through			I know personally
	**Acquaintance**	**Friend**	**Family**	
Senior government official				
Mayor, councilor				
Prestigious professional				
Manager of a large company				
Someone active in a political party or trade union				
Someone active in NGOs				
Someone who knows many people				

27. Could you indicate whether you are a member and/or an active member of the following types of organizations? *(Mark with an X all possible options)*

	Member	Active Member
Religious organizations		
Sports or leisure organizations		
Artistic, musical or educational organizations		
Trade unions		
Political parties		
Environmental organizations		
Professional associations		
Humanitarian or charitable organizations		
Consumer organizations		
Other organizations		

28. Generally, would you say that most people can be trusted or that you should be cautious about others?

Most people can be trusted
You have to be cautious about others

29. How much do you trust the following types of people?

	I do not trust at all	I do not trust much	I trust somewhat	I trust a lot	I am very confident

Family
Neighbors
Friends
Colleagues
People you meet for the first time
People of another religion
Persons of another nationality

Section 7. Happiness and sociodemographic information

30. Thinking about your present life, generally, do you consider yourself a person who is not at all happy, not very happy, somewhat happy, quite happy or very happy?

Not at all happy
Not very happy
Somewhat happy
Quite happy
Very happy

31. If you had to define the level of happiness of the person closest to you, how would you define it? (The person is not at all happy, not very happy, somewhat happy, quite happy or very happy):

Not at all happy
Not very happy
Somewhat happy
Quite happy
Very happy

32. In general, concerning current events, to what extent are you satisfied or dissatisfied with your life? To answer, use the scale from 1 (totally unsatisfied) to 10 (totally satisfied):

33. Age:

34. Gender:

Female
Male

35. Do you have children?

Yes
No
If yes, **how many children do you have?**

36. Educational level attained:

Baccalaureate
Vocational training
University
Master’s degree
Doctorate
Another one:

37. Occupation:

Early childhood education teacher
Primary school teacher
Secondary school teacher
Adult education teacher
Advisor
Other (please specify):

38. On a political ideology scale from left (1) to right (10), where do you see yourself?

Left ☐ ☐ ☐ ☐ ☐ ☐ ☐ ☐ ☐ ☐ Right
1 2 3 4 5 6 7 8 9 10

39. How would you define yourself from a religious point of view?

Religious
Agnostic
Atheist
Other (please specify):

39.1. If you are a religious, what is your religion?

Catholic
Evangelist
Jewish
Muslim
Orthodox
Buddhist
Protestant

40. In addition, could you tell us how often you attend religious ceremonies or practices?

Never or practically never
Less than once a year
Once a year
Only on special occasions
Monthly
Weekly
More than once a week
Daily
